# Factors driving norovirus transmission in long-term care facilities: A case-level analysis of 107 outbreaks

**DOI:** 10.1016/j.epidem.2023.100671

**Published:** 2023-01-18

**Authors:** Yangping Chen, Benjamin A. Lopman, Aron J. Hall, Anita K. Kambhampati, Lynn Roberts, Jordan Mason, Kelley Vilen, Ellen Salehi, Angela Fraser, Carly Adams

**Affiliations:** a Department of Epidemiology, Rollins School of Public Health, Emory University, 1518 Clifton Rd, Atlanta, GA 30322, USA; b Division of Viral Diseases, National Center for Immunization and Respiratory Diseases, Centers for Disease Control and Prevention, 1600 Clifton Rd, Atlanta, GA 30333, USA; c Division of Public Health, Wisconsin Department of Health Services, 1 W Wilson St, Madison, WI 53703, USA; d Foodborne Disease Unit, Minnesota Department of Health, 625 Robert St N, St Paul, MN 55164, USA; e Bureau of Infectious Diseases, Ohio Department of Health, 246 N High St, Columbus, OH 43215, USA; f Department of Food, Nutrition and Packaging Science, Clemson University, 105 Sikes Hall, Clemson, SC 29634, USA

**Keywords:** Norovirus, Outbreaks, Long-term care facilities, Transmission dynamics, Risk factors, Reproduction number

## Abstract

Norovirus is the most common cause of gastroenteritis outbreaks in long-term care facilities (LTCFs) in the United States, causing a high burden of disease in both residents and staff. Understanding how case symptoms and characteristics contribute to norovirus transmission can lead to more informed outbreak control measures in LTCFs. We examined line lists for 107 norovirus outbreaks that took place in LTCFs in five U.S. states from 2015 to 2019. We estimated the individual effective reproduction number, ${\text{R}}_{i}$, to quantify individual case infectiousness and examined the contribution of vomiting, diarrhea, and being a resident (vs. staff) to case infectiousness. The associations between case characteristics and ${\text{R}}_{i}$ were estimated using a multivariable, log-linear mixed model with inverse variance weighting. We found that cases with vomiting infected 1.28 (95 % CI: 1.11, 1.48) times the number of secondary cases compared to cases without vomiting, and LTCF residents infected 1.31 (95 % CI: 1.15, 1.50) times the number of secondary cases compared to staff. There was no difference in infectiousness between cases with and without diarrhea (1.07; 95 % CI: 0.90, 1.29). This suggests that vomiting, particularly by LTCF residents, was a primary driver of norovirus transmission. These results support control measures that limit exposure to vomitus during norovirus outbreaks in LTCFs.

## 1. Introduction

Norovirus is the leading cause of acute gastroenteritis globally and in the United States (Liao et al., 2021; Burke et al., 2021). In the U.S., more than 2000 outbreaks of norovirus are reported to the Centers for Disease Control and Prevention (CDC) each year, the majority of which take place in long-term care facilities (LTCFs) (Calderwood et al., 2021). Due to the high infectivity of norovirus (Evans et al., 2002), combined with the congregate nature of LTCFs and frequent and prolonged contact between LTCF residents and staff (Petrignani et al., 2015), norovirus can spread rapidly in LTCFs, resulting in high attack rates among both residents and staff (Calderwood et al., 2021).

Norovirus outbreaks can result in substantial morbidity and mortality in LTCFs (Calderwood et al., 2021). LTCF residents are generally older adults, often with underlying medical conditions (Moore et al., 2014), and are therefore at greater risk for severe illness and death from norovirus infection (Chen et al., 2017; Mattner, 2006). While norovirus gastroenteritis is generally self-limiting and typically resolves without medical attention, older individuals (> 65 years) have the greatest risk for norovirus-associated severe illness and death, accounting for 90 % of all norovirus-associated mortality in the United States (Hall et al., 2012).

Because there is currently no licensed norovirus vaccine, effective prevention and control measures are critical to minimize norovirus transmission in LTCFs. While norovirus can be transmitted through multiple routes, including contaminated food, water and surfaces, the vast majority of LTCF norovirus outbreaks in the U.S. are spread via person-to-person transmission (Calderwood et al., 2021). Both stool and vomit are infectious, with aerosol dispersal of vomiting spray reportedly able to lead to a wide radius of surrounding environmental contamination (Evans et al., 2002). While asymptomatic infections can occur, symptomatic individuals have been found to be the main drivers of transmission in LTCF outbreaks (Sukhrie et al., 2012). Current outbreak control measure guidelines for U.S. LTCFs are based on general infection control principles, including enhanced environmental cleaning, enhanced hand hygiene, and case isolation (Chen et al., 2017). However, the effectiveness of these measures in controlling norovirus under real-world conditions is not well quantified (Iturriza-Gómara and Lopman, 2014).

By examining transmission patterns and factors associated with increased transmissibility, we can improve the evidence base for interventions aimed at reducing the burden of norovirus outbreaks in LTCFs. In a previous small study of six outbreaks, we (a subset of present authors) found that vomiting among LTCF residents was a primary driver of transmission (Adams et al., 2020). In this present study, we use a larger dataset of 107 U.S. LTCF norovirus outbreaks to examine and quantify risk factors for norovirus infectiousness, with the ultimate goal of further informing evidence-based control measures in this setting.

## 2. Materials and methods

### 2.1. Data source

We requested data on norovirus outbreaks that occurred in LTCFs (nursing homes, skilled nursing facilities, and assisted living facilities) with a first illness onset date between December 1, 2012 and May 1, 2019 from five state health departments that were able to provide complete line lists from a subset of norovirus outbreaks that had occurred in LTCFs in their respective states during this time frame. As this was a convenience sample, the number of outbreaks that occurred during each year may not reflect the true burden of norovirus outbreaks that occurred in LTCFs in each state. Data were provided in the form of de-identified line lists that included the following case-specific information: 1) date of symptom onset, 2) the presence/absence of individual symptoms, including vomiting and diarrhea, 3) whether the case was a resident or staff, and 4) demographic information (e.g., gender and age). Line lists from only those outbreaks that captured data on all identified cases, including both resident and staff cases, throughout the entirety of an outbreak were included in this analysis. Outbreaks were either confirmed (i.e., ≥ 2 laboratory-confirmed cases) or suspected (i.e., epidemiological evidence and/or < 2 laboratory-confirmed cases) to be caused by norovirus (Dewey-Mattia et al., 2018; Forms and guidance for people who complete outbreak reports for submission to CDC NORS). Five state health departments (Minnesota, New Mexico, Ohio, South Carolina, and Wisconsin) provided data for a total of 108 outbreaks; one outbreak was excluded from all analyzes due to substantial missingness of symptom onset dates (45 % of cases).

### 2.2. Individual effective reproduction number, $R_{i}$, estimation

In this analysis, our primary outcome of interest was individual case infectiousness, which we quantified by the individual effective reproduction number, ${\text{R}}_{i}$, defined as the number of secondary cases that an individual, i, generates in an outbreak. Case infectiousness may be affected by several different factors, including contact patterns, symptomology, and viral shedding. We estimated ${\text{R}}_{i}$ using the Wallinga- Teunis (WT) method, which uses a maximum likelihood algorithm based on transmission probability between any pair of cases in an outbreak. This method is described in detail elsewhere (Wallinga and Teunis, 2004; Teunis et al., 2013). Briefly, it uses the difference in symptom onset dates between cases and the probability distribution of the serial interval, or the time between symptom onset in primary cases and the secondary cases they generate (Fine, 2003), to calculate the relative likelihood that cases with earlier symptom onset dates infected cases with later symptom onset dates. The relative likelihood that case i infected any other case j is normalized and summed to estimate ${\text{R}}_{i}$, and corresponding 95 % confidence intervals are generated after multiple simulations. Cases cannot infect other cases with the same or earlier symptom onset dates, and cases with the same symptom onset date will have the same ${\text{R}}_{i}$ and corresponding 95 % confidence intervals. $R_{i}$ from cases with symptom onset dates on the last day of an outbreak will always equal 0, as these cases did not generate any known secondary cases. We used a gamma probability distribution for the serial interval with a mean of 3.6 days and standard deviation of 2.0 days, which was derived from a previous study of several large norovirus outbreaks in several camps at an international scouting jamboree in Sweden (Heijne et al., 2009). Considering the uncertainty of the probability distribution of the norovirus serial interval in U.S. LTCF outbreaks, we also conducted a sensitivity analysis with the mean serial interval lengths varying between 1.5 and 4.0 days in half day increments.

### 2.3. Risk factor analysis for infectiousness

We used a linear mixed model to examine associations between individual case characteristics and ${\text{R}}_{i}$. Because the distribution of ${\text{R}}_{i}$ was right-skewed, we used the natural log of ${\text{R}}_{i}$ as the dependent variable. In order to retain cases with $R_{i}=0$ in the regression analysis, we added a small value (0.01) to these cases’ ${\text{R}}_{i}$ to perform the log transformation. As a sensitivity analysis, we also examined all associations with such cases excluded. We selected presence of vomiting (yes or no), presence of diarrhea (yes or no), and being a resident or staff as our main exposure variables. We also examined evidence for an interaction between vomit and diarrhea.

The linear mixed model included a random intercept for outbreak, which accounted for clustering induced by correlation of ${\text{R}}_{i}$ within the 107 outbreaks. To incorporate the uncertainty of each ${\text{R}}_{i}$ estimate, we used inverse variance weighting, with the weight of each ${\text{R}}_{i}$ equal to the inverse variance of the estimated ${\text{R}}_{i}$. The linear mixed model is specified as follows:

$$\text{log}R_{ij}=\beta_{0}+b_{0i}+\beta_{1}Diarrhea_{ij}+\beta_{2}Vomiting_{ij}+\beta_{3}Resident_{ij}+e_{ij}$$

where $\text{log}\;R_{ij}$ represents the estimated natural log of the effective reproduction number of the jth case from the ith outbreak, $b_{0i}$ represents the random intercept for the ith outbreak, and $e_{ij}$ represents residual heterogeneity of the jth case from the ith outbreak not explained by the model. The residual heterogeneity, $e_{ij}$, and random intercept, $b_{0i}$, were assumed to be independent and identically distributed with mean zero and their respective variances. Cases from the same outbreak were assigned the same random effect, whereas cases from different outbreaks were assumed to be independent. Final coefficient estimates and 95 % confidence intervals were exponentiated to show the relationships between average $R_{i}$ (rather than $\text{log}\;R_{i}$) and the variables in the model. All statistical analyses were performed using the *EpiEstim* (Cori et al., 2013) and *metafor* (Viechtbauer and Viechtbauer, 2010) packages in R v.4.0.3. This model was adapted from a previous study (Adams et al., 2020). Furthermore, we visually examined associations between symptoms/characteristics and $R_{i}$ using histograms, showing the number of cases by $R_{i}$ stratified by symptom/characteristic.

We conducted three secondary analyses. First, we examined whether index cases (i.e., cases with symptom onset on day 1 of an outbreak) were more likely than non-index cases to have specific symptoms/characteristics. We used a multivariable logistic regression model with vomiting, diarrhea, and resident vs. staff as the independent variables and index case (yes or no) as the dependent variable. All cases were included in this analysis. Second, we examined the relative infectiousness of index cases to non-index cases by fitting a bivariable linear mixed model, with $\text{log}\;R_{i}$ as the dependent variable and index case (yes or no) as the independent variable. To minimize the impact of control measures and accumulation of acquired immunity on $R_{i}$, we only included cases with illness onset within the first four days of the outbreak in this analysis. Lastly, to examine whether cases with specific symptoms/characteristics occurred earlier in outbreaks, we used a linear mixed multivariable model to examine the associations between symptom onset day and vomiting, diarrhea, and resident vs. staff cases.

Because this study involved the use of de-identified data that had already been collected through routine public health surveillance, Emory University and CDC Institutional Review Boards (IRB) determined that IRB review was not required.

## 3. Results

### 3.1. Outbreak characteristics

A total of 107 outbreaks and 3363 cases were included in the analyzes. The median number of cases per outbreak was 27 (interquartile range [IQR]: 18.5, 37) and the median outbreak length (difference in symptom onset dates between the first ill case and the last ill case) was 13 days (IQR: 8.5, 18.5) (Table 1). The majority of cases were female (71 %) and over 65 years old (81 %), with a mean age of 76.6 years (Table 2). Sixty-six percent of cases reported vomiting, 78% reported diarrhea, and 63 % were LTCF residents. The majority of our sample of outbreaks occurred from 2017 to 2019 (94 %) and during the winter (December–February) months (59 %). Of 89 outbreaks with genotype information, 56 (63 %) were caused by norovirus GII.4.

### 3.2. Outbreak transmission patterns based on case characteristics/ symptoms

**Fig. 1.**
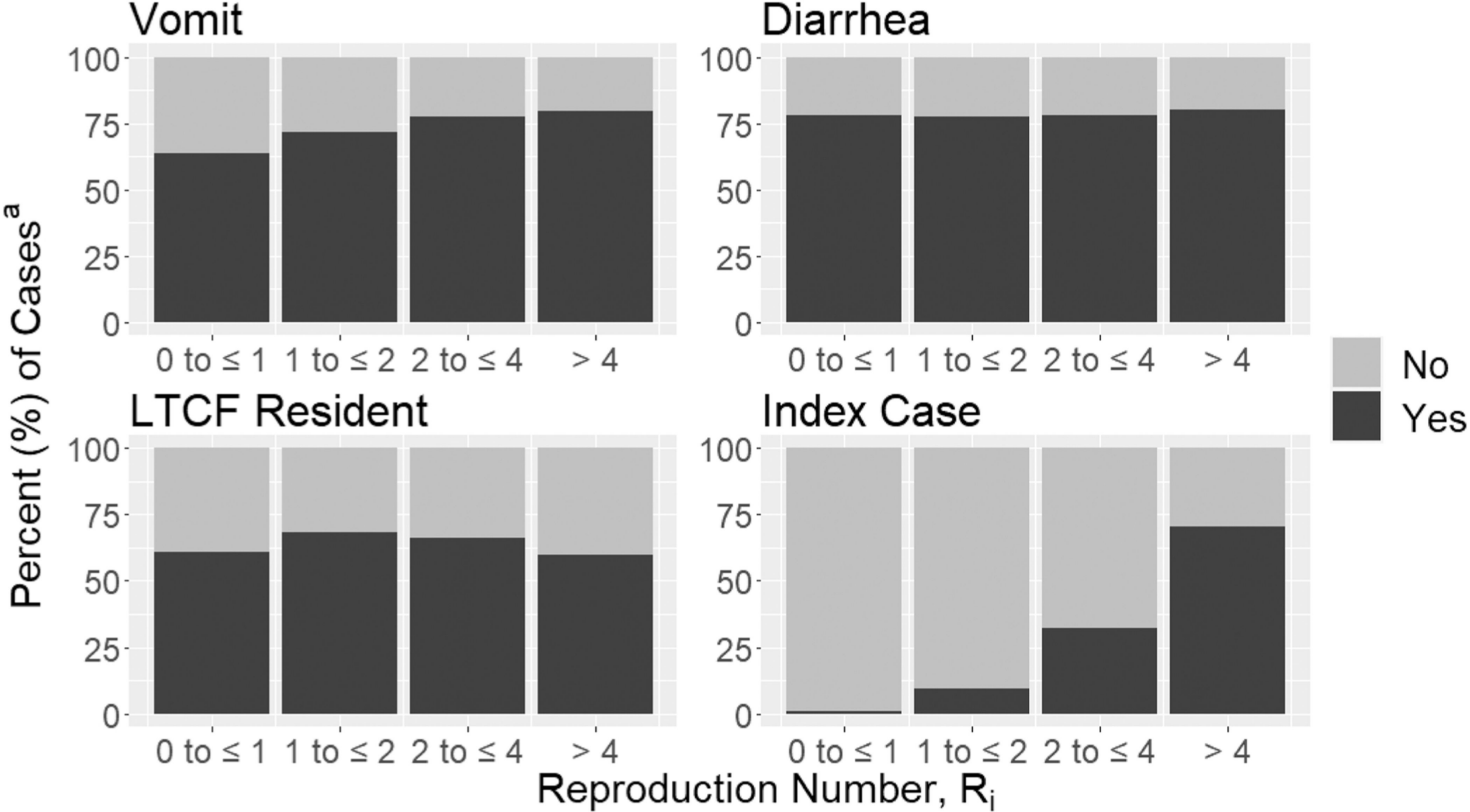
Percent of cases with symptom/characteristic by categorized ${\text{R}}_{i}$ value. Abbreviations: long-term care facility, LTCF, a) Cases with missing information were excluded from percentage calculations.

We found that $R_{i}$ estimates for index cases were substantially larger (median $R_{i}=2.16$ [IQR: 1.13, 4.57]) than those for non-index cases (median $R_{i}=0.59$ [IQR: 0.35, 1.02]) for all outbreak days. Of cases who were highly infectious (i.e., $R_{i}>4$), 70.2 % were index cases (Fig. 1). Similarly, $R_{i}$ values were positively correlated with the proportion of cases who vomited. When $R_{i}$ values were between 0 and 2, $R_{i}$ was also positively correlated with the proportion of resident cases. However, there was no correlation between $R_{i}$ and the proportion of cases with diarrhea.

### 3.3. Risk factors for norovirus transmission

**Fig. 2.**
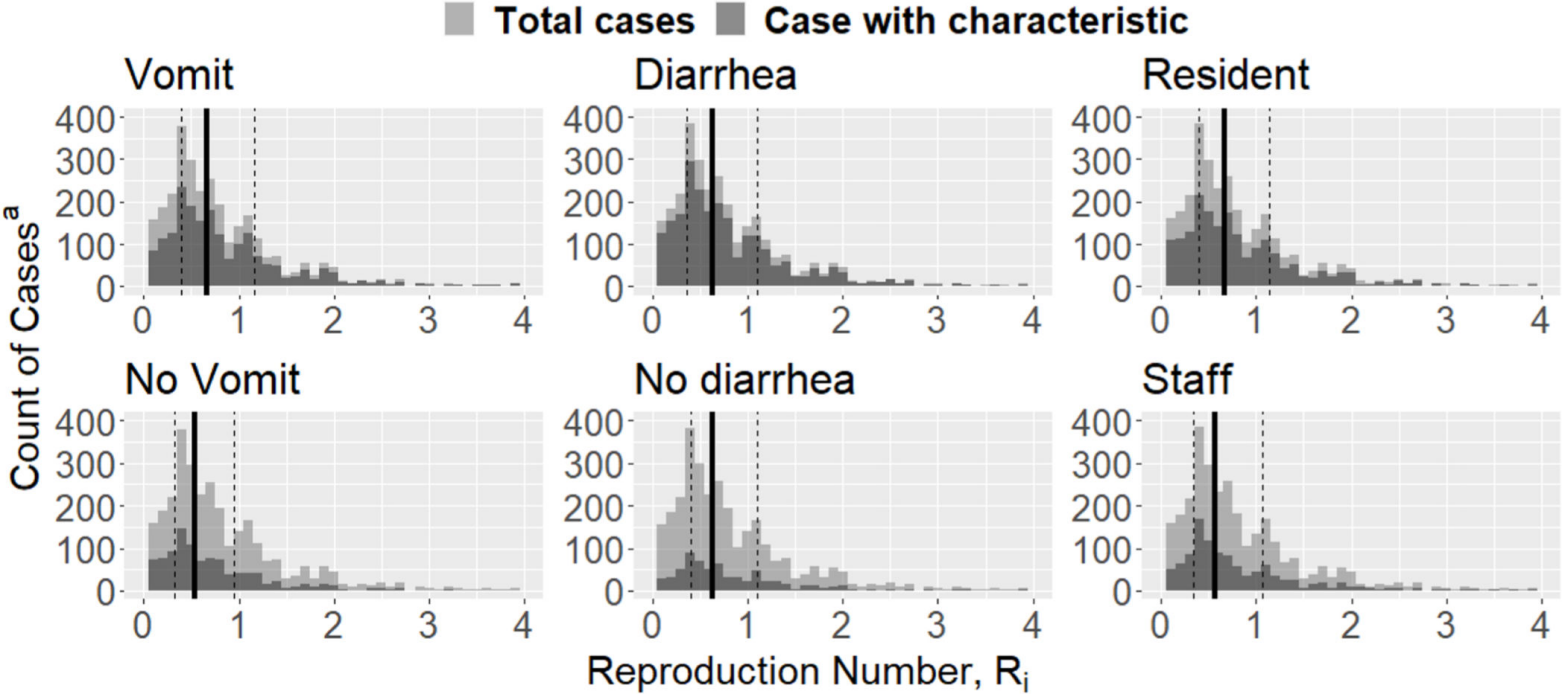
Number of cases with symptom/characteristic by ${\text{R}}_{i}$ value^b^. a) Cases with missing information were excluded from count calculations. b) Solid lines represent the median ${\text{R}}_{i}$ and dashed lines represent the 25th and 75th quantiles.

The median $R_{i}$ among cases with vomiting (0.67, IQR: 0.40–1.16) was slightly larger than that among cases without vomiting (0.53, IQR: 0.32–0.95), however interquartile ranges (IQRs) largely overlapped (Fig. 2). Similarly, the median $R_{i}$ among resident cases (0.67, IQR: 0.39–1.14) was slightly larger than that among staff cases (0.57, IQR: 0.34–1.07), but IQRs again largely overlapped. There was no difference in median $R_{i}$ between cases with and without diarrhea (median ${\text{R}}_{i}=0.63$ [IQR: 0.36–1.10] and 0.64 [IQR: 0.41–1.10], respectively).

In regression analyses, 167 (5 %) cases were excluded due to missing outcome and/or exposure information, resulting in a total of 3196 cases included in the analysis. We found that cases who vomited infected 1.35 (95 % CI: 1.18, 1.54) times the number of secondary cases compared to cases without vomiting, and LTCF residents infected 1.31 (95 % CI: 1.16, 1.48) times the number of cases compared to cases among staff. Cases with and without diarrhea were equally infectious (1.08; 95 % CI: 0.91–1.29). The average/median estimates of $R_{i}$, which is the average/median number of subsequent cases infected in all scenarios, are summarized below (Table 3):

Considering a large proportion of outbreak data were from Wisconsin (67.3 %), we also did the subgroup regression analysis on 1) only the outbreaks that were reported from Wisconsin, and 2) outbreaks from all other states except Wisconsin to ensure these two regression outputs were consistent with the main results. Both the regression outputs were consistent with the analyzes including all outbreak data. Also, to examine potential differences in results by state, we added US state as a fixed effect in the final model (including all outbreak data). The regression coefficients for US state were not significant (set Minnesota as reference, β for New Mexico is (0.90; 95 % CI: 0.49, 1.64), β for Ohio is (1.28; 95 % CI: 0.35, 4.75), β for South Carolina is (0.86; 95 % CI: 0.43, 1.71), β for Wisconsin is (0.98; 95 % CI: 0.63, 1.55), and the estimates for vomit, diarrhea and resident vs. staff did not change meaningfully.

In sensitivity analyses excluding cases with $R_{i}=0$, results were similar for associations between $R_{i}$ and vomiting (1.30; 95 % CI: 1.17, 1.45) and diarrhea (1.03; 95 % CI: 0.89, 1.18), and slightly attenuated for residents vs. staff (1.16; 95 % CI: 1.05, 1.28). Lastly, we found no evidence of an interaction between vomiting and diarrhea.

**Fig. 3.**
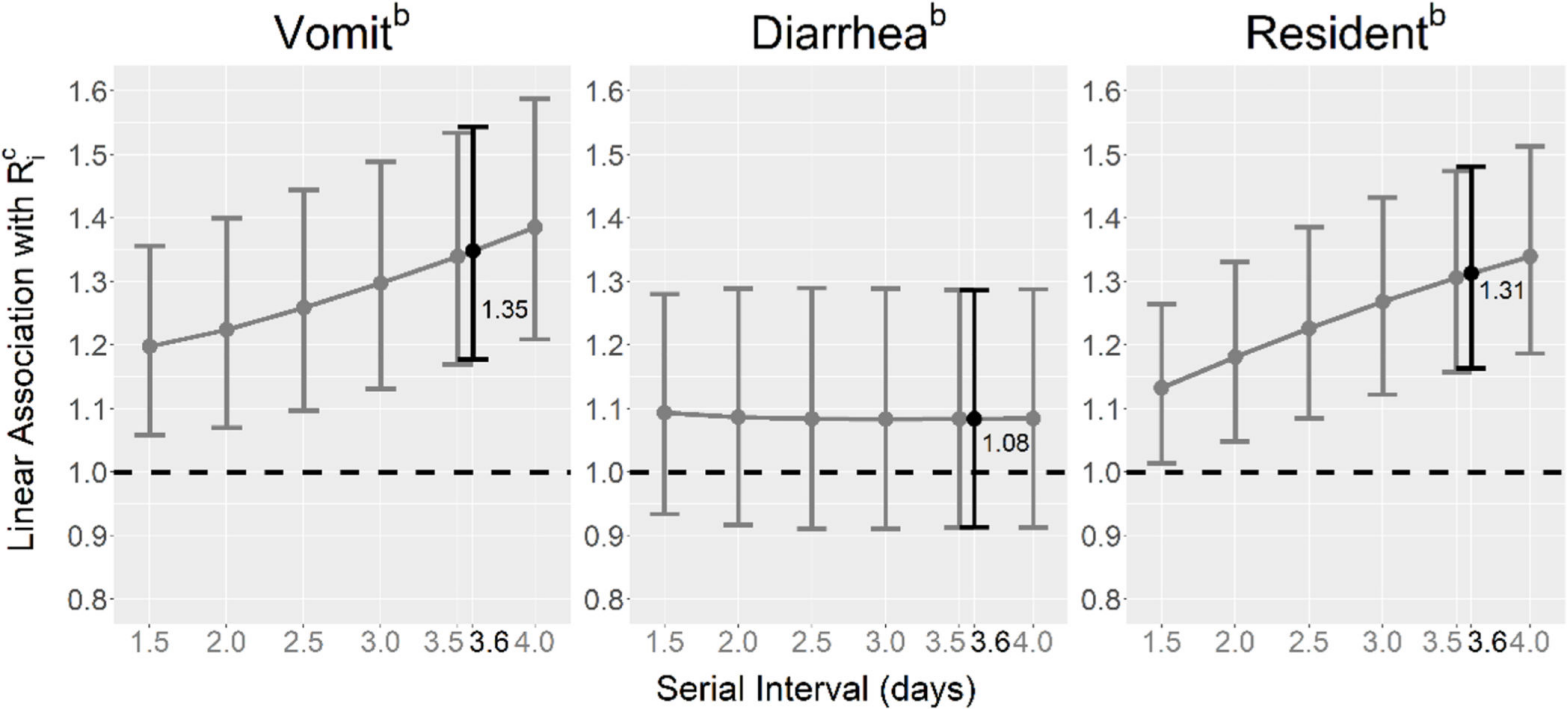
Associations between individual reproduction numbers, $R_{i}$, and symptoms/characteristics of norovirus cases by serial interval length^a^. a) The serial interval length used in the final regression analysis is shown in black. b) Associations were estimated using a multivariable linear mixed regression model with a log-transformed outcome variable ($R_{i}$), inverse-variance weighting, a random intercept for outbreak number, and the following dichotomous predictor variables: vomiting (vs. no vomiting), diarrhea (vs. no diarrhea), and resident (vs. staff). c) Estimates from the model were exponentiated.

We found that these results were robust to assumptions about the serial interval length used in $R_{i}$ calculations (Fig. 3). Associations between vomiting and resident vs. staff and $R_{i}$ increased in magnitude as the serial interval length increased. The association between diarrhea and $R_{i}$ remained approximately null for all serial interval lengths.

### 3.4. Comparison of index cases to non-index cases

Among all cases, index cases were more likely to have vomited (73.1 %) compared to non-index cases (64.8 %), whereas the percent of cases with diarrhea was similar between index and non-index cases (75.8 % and 76.8 %, respectively). The percent of index cases that were residents (65.3 %) was similar to that which were staff (61.0 %). We found that index cases were more likely to have vomiting (OR = 1.65; 95 % CI: 1.18, 2.35) and residents (OR = 1.54; 95 % CI: 1.13, 2.14) compared to non-index cases (including all outbreak days). We found no association between being an index case and having diarrhea (OR = 1.07; 95 % CI: 0.76, 1.54). When considering only cases with symptom onset on outbreak days 1–4, we found that index cases were considerably more infectious than non-index cases, infecting 2.23 (95 % CI: 1.77, 2.79) times the number of secondary cases than non-index cases.

### 3.5. Associations between case characteristics and outbreak day

**Fig. 4.**
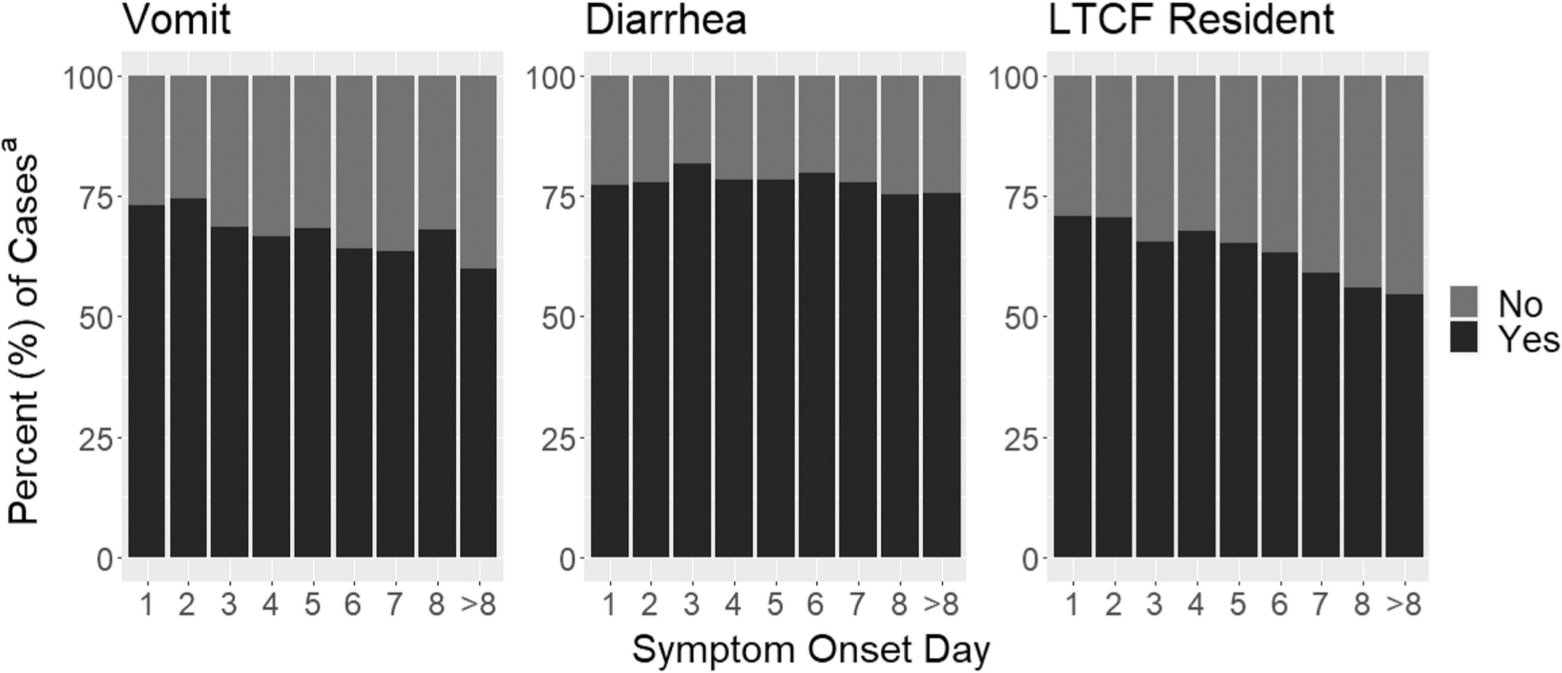
Percentages^a^ of cases with symptom/characteristic by day of illness onset^b^. Abbreviations: long-term care facility, LTCF, a) Cases with missing information were excluded from percentage calculations. b) Symptom onset day refers to the outbreak day on which cases had symptom onset, excluding days on which no cases had symptom onset.

Lastly, in our multivariable linear mixed model examining the associations between symptom onset day and vomit, diarrhea, and resident vs. staff, we found that cases who vomited had illness onset 1.01 (95 % CI: 0.65, 1.37) days earlier in an outbreak compared to cases who did not vomit; cases among LTCF residents occurred 1.13 (95 % CI: 0.78, 1.47) days earlier in an outbreak compared to cases among LTCF staff; and cases with diarrhea occurred 0.46 (95 % CI: 0.05, 0.87) days earlier in an outbreak compared to cases without diarrhea. In general, the proportion of cases that vomited and the proportion that were LTCF residents decreased as outbreaks progressed, whereas the proportion of cases that had diarrhea remained about the same (Fig. 4).

## 4. Discussion

This study used a large dataset of 107 LTCF norovirus outbreaks from five states reported between 2015 and 2019. To our knowledge, this is the largest dataset used for a norovirus transmission study. We quantified individual case infectiousness for norovirus outbreaks by inferring who infected whom from symptom onset dates and the norovirus serial interval distribution, and then examined individual risk factors for infectiousness and temporal changes in infectiousness, leading to several important findings. We found that first, vomiting likely plays an important role in norovirus transmission in U.S. LTCFs. Second, LTCF residents are more infectious than staff. Lastly, outbreaks tend to start with one or more index cases who are considerably more infectious than subsequent, non-index cases. These results are based on a large dataset with information on 107 LTCF norovirus outbreaks and more than 3000 cases.

This study supports results from a previous study in which authors found that cases with vomiting can infect more secondary cases than cases without vomiting and residents can infect more secondary cases than staff in U.S. LTCF norovirus outbreaks (Adams et al., 2020). However, while the previous study also found that cases with diarrhea were slightly more infectious than cases without diarrhea, we found no association between diarrhea and increased infectiousness. This discrepancy may be due to the difference in sample size, with the previous study including information from only 6 norovirus outbreaks and 208 cases. With our much larger dataset, we found that diarrhea does not appear to play as important a role in transmission.

Exposure to vomit has been identified as a risk factor for norovirus infection and transmission in LTCFs (Petrignani et al., 2015). By testing air samples close to hospitalized patients with norovirus infection, one study found that recent vomiting (within 3 h since the last vomiting episode) was a major source of airborne norovirus (Alsved et al., 2020). The study also found that there was no association between positive air samples and time since the last instance of diarrhea, indicating that diarrhea may play less of a role in transmission than vomiting due to lack of aerosolization.

We also found that residents are more infectious than staff in LTCF norovirus outbreaks. A previous study found that initial fecal viral loads in norovirus-infected residents were higher than those in staff during a nursing home norovirus outbreak, indicating that increased resident infectiousness may be due to increased viral shedding (Lai et al., 2013). Furthermore, when staff exhibit symptoms, current infection control guidelines require they be excluded from work for a minimum of 48 h after the resolution of symptoms, during which time they cannot transmit infection within the LTCF (Norovirus Guidelines for Healthcare Settings). Residents, on the other hand, require continued care, and possibly even more care, after developing symptoms, so they can potentially transmit norovirus throughout the duration of their illness.

Lastly, we found that index cases were more infectious than non- index cases. There are several potential explanations for this, which were noted in the previous study (Adams et al., 2020). First, a high level of infectiousness from inherent case characteristics (e.g., high frequency of contact with others or increased shedding) and/or symptomology (e. g., vomiting) may be required for index cases to initiate and maintain an outbreak. For instance, if a case is not highly infectious, transmission may end with that case and an outbreak will not occur. Second, there is a natural decrease in the reproduction number, ${\text{R}}_{i}$, as outbreaks progress and more individuals become ill and later immune, which may result in index cases having inflated ${\text{R}}_{i}$’s compared to subsequent cases in the same outbreak. Third, the implementation of control measures after the occurrence of an index case could result in reduced ${\text{R}}_{i}$ in cases later in an outbreak. Fourth, there is a certain level of population genetic immunity expected, which make some people have low susceptibility to norovirus. Lastly, index cases may have intrinsic case characteristics, like vomiting or being LTCF residents, that increases their infectiousness. Most likely, the increased infectiousness in index cases is due to some combination of all four possible explanations.

We note a number of limitations in our study. First, we assumed that line lists were complete (i.e., no missing cases) and that asymptomatic cases did not contribute to transmission. However, it’s possible that cases were missing from line lists, particularly if they occurred earlier in the outbreak or were asymptomatic, which could result in an inflated estimate of index case infectiousness. It is also possible for asymptomatic cases to contribute to norovirus transmission (Teunis et al., 2015), however symptomatic cases are estimated to be the main drivers of transmission in healthcare settings (Sukhrie et al., 2012). Second, we did not have detail on type of LTCF setting and therefore could not consider the heterogeneity of LTCF settings (e.g., nursing home, skilled nursing facilities, assisted living, etc.), which may impact transmission patterns of norovirus due to different infrastructures, and staff-resident contact intensities, and mobility of residents. We attempted to gather more information on outbreak setting using data from the public health departments from the five states, however few outbreak reports contained this information and we were unable to examine it further. Third, we used a serial interval derived from several large norovirus outbreaks in several camps in Sweden (Heijne et al., 2009), which may not be generalizable to norovirus outbreaks in U.S. LTCFs. However, results were generally robust in sensitivity analyses, in which shorter and longer serial intervals were used to estimate ${\text{R}}_{i}$. Fourth, we did not consider heterogeneities in staff roles when examining case infectiousness, and staff who provide direct care to residents (e.g., certified nursing assistants and registered nurses) may be more infectious than staff who provide little or no direct patient care (e.g., administrative staff). We did not have information on staff role or time spent in facility and were therefore unable to examine these further. Fifth, we assumed that all cases had the same opportunity to interact with other residents or staff in an outbreak, but it is unlikely that all cases had equal opportunity for exposure, since facilities may have separate living and dining facilities, and may also implement precautions such as isolation of ill residents once outbreaks begin. Sixth, we did not consider the effect of different norovirus genotypes on symptoms, infectivity and transmission of norovirus. Lastly, we assumed that cases could not infect cases with the same or earlier symptom onset dates. Because norovirus has a short incubation period (Devasia et al., 2015), and because incubation periods can vary, it is possible, although unlikely, for the serial interval to be non-positive.

Because there is currently no publicly available vaccine or specific antiviral treatment, general infection control measures are the mainstay for curtailing norovirus transmission in LTCFs. Our study provides support for measures that target cases who vomit, particularly resident cases who vomit, and that limit exposure to vomitus. Rapid response to vomiting events, including cleaning and disinfecting contaminated environments with an EPA-registered disinfectant and isolating cases who vomit, may help to reduce the size and duration of norovirus outbreaks in U.S. LTCFs. Additionally, quickly identifying and isolating early symptomatic cases, including index cases, may substantially reduce transmission. Future studies should consider collecting detailed data on LTCF norovirus outbreak control measures, including which control measures were implemented and when, to evaluate the effectiveness of specific control measures on reducing norovirus transmission.

## 5. Conclusions

In this study of 107 norovirus outbreaks in U.S. LTCFs, vomiting, particularly by LTCF residents, was a primary driver of norovirus transmission. These results support control measures that limit exposure to vomitus during norovirus outbreaks in LTCFs.

## Supplementary Material

Supplementary Data

## Figures and Tables

**Table 1:** Summary of long-term care facility norovirus outbreaks from five US states, 2015–2019.

Variable	All outbreaks (No. = 107)

**Total Cases**	
(No.)	3363
(Median (IQR))^a^	27 (18.5, 37)
(Mean (SD))^a^	31.4 (21.9)
**Outbreak Length (days)**	
(Median (IQR))^a^	13 (8.5, 18.5)
(Mean (SD))^a^	14.5 (8.5)
**State** (No. (%))^b^	
Wisconsin	72 (67.3)
New Mexico	17 (15.9)
Minnesota	11 (10.3)
South Carolina	6 (5.6)
Ohio	1 (0.9)
**Year** (No. (%))^b^	
2015	4 (3.7)
2016	2 (1.9)
2017	20 (18.7)
2018	45 (42.1)
2019	36 (33.6)
**Season** (No. (%))^b^	
Spring (March–May)	38 (35.5)
Summer (June–August)	2 (1.9)
Fall (September–November)	4 (3.7)
Winter (December–February)	63 (58.9)
**Norovirus Genotype** (No. (%))^b,c^	
GII.4	56 (56.6)
GII, Non-GII.4	18 (18.2)
GII with unknown genotype	6 (6.0)
GI with known or unknown genotype	19 (19.2)

Abbreviations: long-term care facility, LTCF; number, No.; interquartile range, IQR; standard deviation, SD.

a Median (IQR) and mean (SD) per outbreak.

b Number (%) and mean (SD) across all outbreaks. Percentages were calculated excluding cases with missing information.

c Percentages were calculated excluding outbreaks with missing genogroup information.

**Table 2:** Case characteristics from all long-term care facility norovirus outbreaks from five US states, 2015–2019.

Variable	Total	Residents	Staff

**Total Cases**			
(No.)	3363	2061	1229
(Median (IQR))^a^ (Mean (SD))^a^	27 (18.5, 37) 31.4 (21.9)	18 (11, 24) 20.2 (14.2)	11 (6, 17) 13.1 (10.1)
**Cases with Diarrhea** (No. (%))^b^	2578 (77.9)	1621 (79.2)	902 (75.6)
**Cases with Vomiting** (No. (%))^b^	2198 (66.4)	1239 (60.8)	929 (77.7)
**Cases with both Diarrhea and Vomiting** (No. (%))^b^	1508 (46.0)	838 (41.3)	656 (55.7)
**Gender**			
Female (No. (%))^b^	1637 (71.3)	1005 (64.0)	600 (92.0)
Male (No. (%))^b^	659 (28.7)	566 (36.0)	52 (8.0)
**Age in Years** (Mean (SD))^b^	76.6 (18.1)	81.9 (10.8)	38.3 (15.1)

Abbreviations: long-term care facility, LTCF; number, No.; interquartile range, IQR; standard deviation, SD.

a Median (IQR) and mean (SD) per outbreak.

b Number (%) and mean (SD) across all outbreaks. Percentages were calculated excluding cases with missing information.

**Table 3:** Average ${\text{R}}_{i}$ from cases with specific symptom/characteristic from 107 norovirus outbreaks from five US states, 2015–2019.

Vomit	Diarrhea	Resident	Average $R_{i}$	Median $R_{i}$

Yes	Yes	Yes	1.12	0.74
No	Yes	Yes	0.79	0.57
Yes	No	Yes	0.97	0.70
Yes	Yes	No	0.94	0.60
No	No	Yes	0.93	0.44
Yes	No	No	0.85	0.58
No	Yes	No	0.77	0.44
No	No	No	0.86	0.66

## References

[R1] Adams C, , 2020. Quantifying the roles of vomiting, diarrhea, and residents vs. staff in norovirus transmission in U.S. nursing home outbreaks. PLoS Comput. Biol 16. 10.1371/journal.pcbi.1007271 PMC7135310 32210423

[R2] Alsved M, , 2020. Sources of airborne norovirus in hospital outbreaks. Clin. Infect. Dis 70. 10.1093/cid/ciz584 PMC7201413 31257413

[R3] Burke RM, , 2021. Burden of norovirus in the United States, as estimated based on administrative data: updates for medically attended illness and mortality, 2001–2015. Clin. Infect. Dis 73. 10.1093/cid/ciaa438 PMC8112883 32291450

[R4] Calderwood LE, , 2021. Norovirus outbreaks in long-term care facilities in the United States, 2009–2018: a decade of surveillance. Clin. Infect. Dis 10.1093/cid/ciab808. PMC8978331 34523674

[R5] Chen Y, Hall AJ, Kirk MD, 2017. Norovirus disease in older adults living in long-term care facilities: strategies for management. Curr. Geriatr. Rep 6. 10.1007/s13670-017-0195-z PMC5709813 29204334

[R6] Cori A, Ferguson NM, Fraser C, Cauchemez S, 2013. A new framework and software to estimate time-varying reproduction numbers during epidemics. Am. J. Epidemiol 178. 10.1093/aje/kwt133 PMC3816335 24043437

[R7] Devasia T, Lopman B, Leon J, Handel A, 2015. Association of host, agent and environment characteristics and the duration of incubation and symptomatic periods of norovirus gastroenteritis. Epidemiol. Infect 143, 2308–2314. 25483148 10.1017/S0950268814003288 PMC8845055

[R8] Dewey-Mattia D, Manikonda K, Hall AJ, Wise ME, Crowe SJ, 2018. Surveillance for foodborne disease outbreaks – United States, 2009–2015. MMWR Surveill. Summ 67. 10.15585/mmwr.ss6710a1 PMC6061962 30048426

[R9] Evans MR, , 2002. An outbreak of viral gastroenteritis following environmental contamination at a concert hall. Epidemiol. Infect 129. 10.1017/s0950268802007446 PMC2869894 12403111

[R10] Fine PEM, 2003. The interval between successive cases of an infectious disease. Am. J. Epidemiol 158. 10.1093/aje/kwg251 14630599

[R11] Forms and guidance for people who complete outbreak reports for submission to CDC NORS. 〈https://www.cdc.gov/nors/forms.html〉.

[R12] Hall AJ, Curns AT, Clifford Mcdonald L, Parashar UD, Lopman BA, 2012. The roles of clostridium difficile and norovirus among gastroenteritis-associated deaths in the United States, 1999–2007. Clin. Infect. Dis 55. 10.1093/cid/cis386 22491338

[R13] Heijne JCM, , 2009. Enhanced hygiene measures and norovirus transmission during an outbreak. Emerg. Infect. Dis 15. 10.3201/1501.080299 PMC2660689 19116045

[R14] Iturriza-Gómara M, Lopman B, 2014. Norovirus in healthcare settings. Curr. Opin. Infect. Dis 27. 10.1097/QCO.0000000000000094 PMC4154788 25101555

[R15] Lai CC, , 2013. A norovirus outbreak in a nursing home: norovirus shedding time associated with age. J. Clin. Virol 56. 10.1016/j.jcv.2012.10.011 23153821

[R16] Liao Y, , 2021. Global prevalence of norovirus in cases of acute gastroenteritis from 1997 to 2021: an updated systematic review and meta-analysis. Microb. Pathog 161. 10.1016/j.micpath.2021.105259 34687838

[R17] Mattner F, , 2006. Risk groups for clinical complications of norovirus infections: an outbreak investigation. Clin. Microbiol. Infect 12. 10.1111/j.1469-0691.2005.01299.x 16460549

[R18] Moore KL, Boscardin WJ, Steinman MA, Schwartz JB, 2014. Patterns of chronic co-morbid medical conditions in older residents of U.S. nursing homes: differences between the sexes and across the agespan. J. Nutr. Heal. Aging 18. 10.1007/s12603-014-0001-y PMC4099251 24676326

[R19] Norovirus Guidelines for Healthcare Settings. 〈https://www.cdc.gov/infectioncontrol/guidelines/norovirus/index.html 〉.

[R20] Petrignani M, van Beek J, Borsboom G, Richardus JH, Koopmans M, 2015. Norovirus introduction routes into nursing homes and risk factors for spread: a systematic review and meta-analysis of observational studies. J. Hosp. Infect vol. 89. 10.1016/j.jhin.2014.11.015 25601744

[R21] Sukhrie FHA, , 2012. Nosocomial transmission of norovirus is mainly caused by symptomatic cases. Clin. Infect. Dis 54. 10.1093/cid/cir971 22291099

[R22] Teunis P, , 2013. Infectious disease transmission as a forensic problem: who infected whom? J. R. Soc. Interface 10. 10.1098/rsif.2012.0955 PMC3627102 23389896

[R23] Teunis PFM, , 2015. Shedding of norovirus in symptomatic and asymptomatic infections. Epidemiol. Infect 143. 10.1017/S095026881400274X PMC9507237 25336060

[R24] Viechtbauer W, Viechtbauer W, 2010. Conducting meta-analyses in R with the metafor package. J. Stat. Softw 36 (3), 1–48. J. Stat. Softw. 36, (2010).

[R25] Wallinga J, Teunis P, 2004. Different epidemic curves for severe acute respiratory syndrome reveal similar impacts of control measures. Am. J. Epidemiol 160. 10.1093/aje/kwh255 PMC7110200 15353409

