# Supplementary material for: Factors driving norovirus transmission in long-term care facilities: A case-level analysis of 107 outbreaks
Source: Epidemics. Author manuscript; Available in PMC 2024 Sep 11. (PMC11389824; doi:10.1016/j.epidem.2023.100671)
Supplement: Supplementary Data [file NIHMS2011691-supplement-Supplementary_Data.pdf]

Supplemental Table 1: Summary of 106 long-term care facility norovirus outbreaks by state, 2015-2019[a]

	No. of	Case Count		Outbreak Duration	
**State**	**Outbreaks**	**Mean (SD)**	**Median (IQR)**	**Mean (SD)**	**Median (IQR)**
Wisconsin	72	30.6 (19.5)	25 (17, 36)	15.1 (8.1)	14 (9, 19.3)
New Mexico	17	22.1 (9.2)	22 (19, 27)	10.2(9.7)	7 (6, 11)
Minnesota	11	37.4 (12.4)	37 (32, 39)	16.6 (6.9)	16 (11.5, 19)
South Carolina	6	34.8 (14.8)	36.5 (28.3, 44.8)	12.8 (2.7)	12 (12, 12.8)

a) A single outbreak from Ohio was excluded from the table.

Supplemental Figure 1: Epidemic curves[a] and estimated individual reproduction numbers, R_i, for all outbreaks

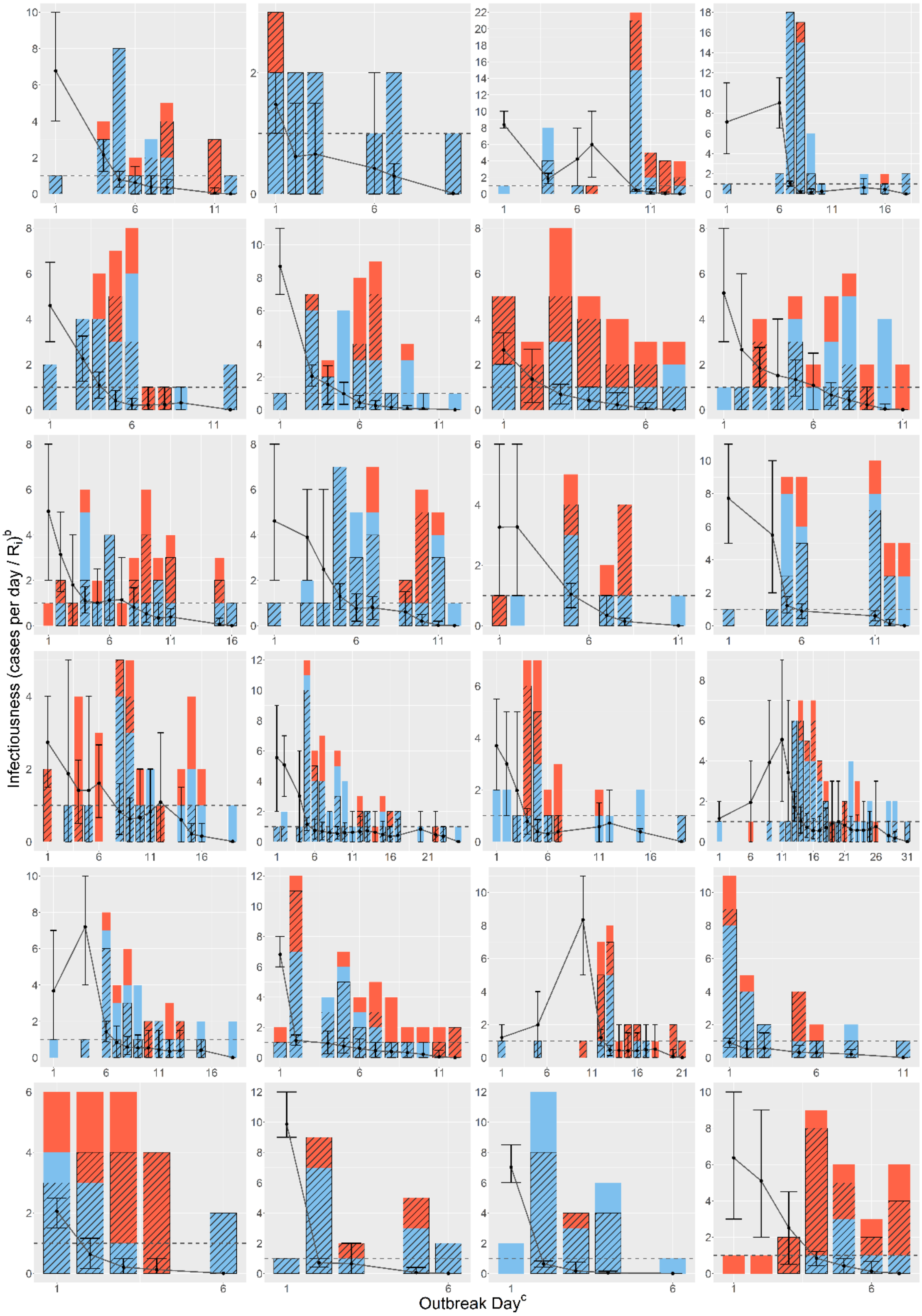

Infectiousness (cases per day / R_i)[b]
Outbreak Day[c]

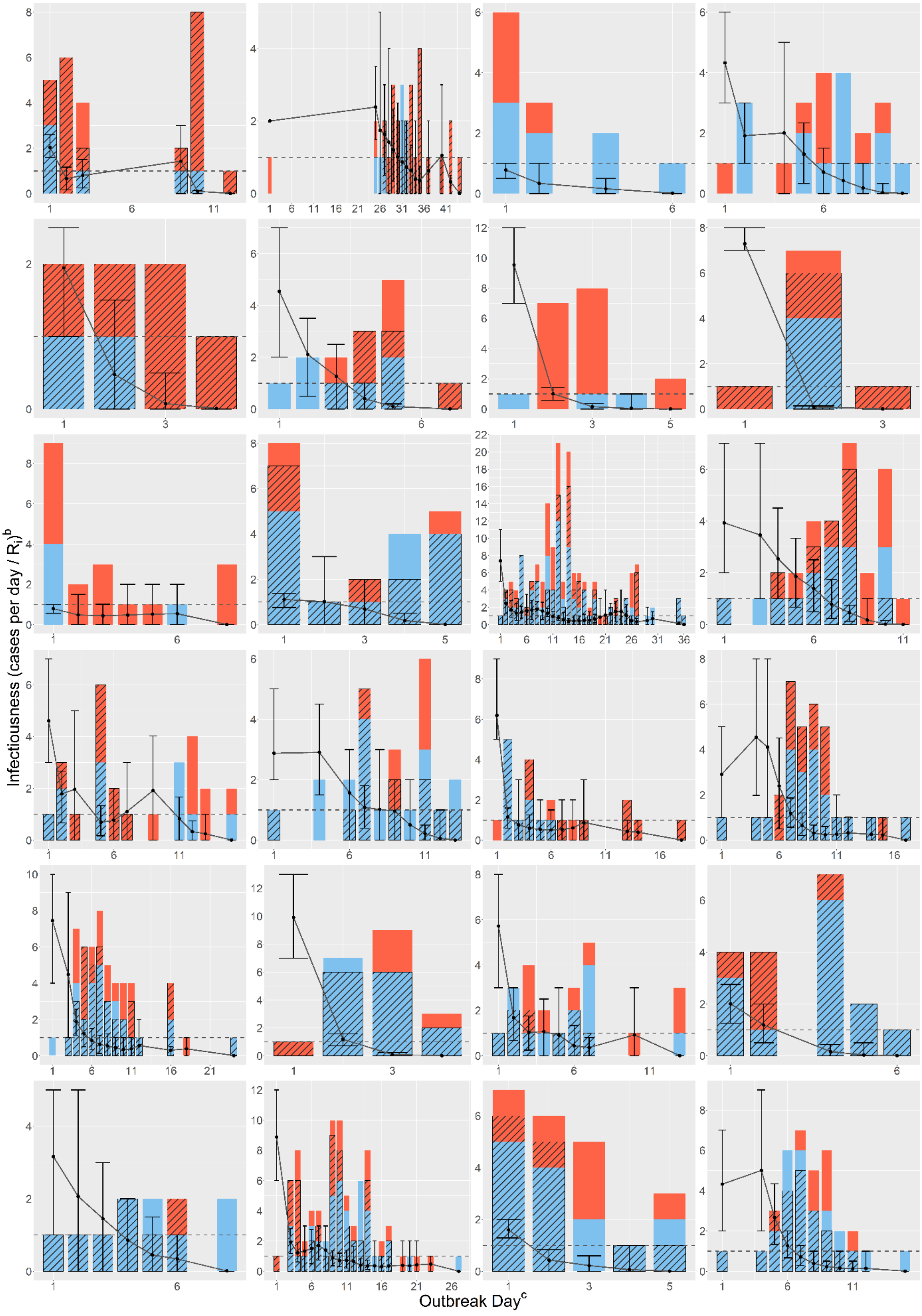
Infectiousness (cases per day / R_i)^b
Outbreak Day^c

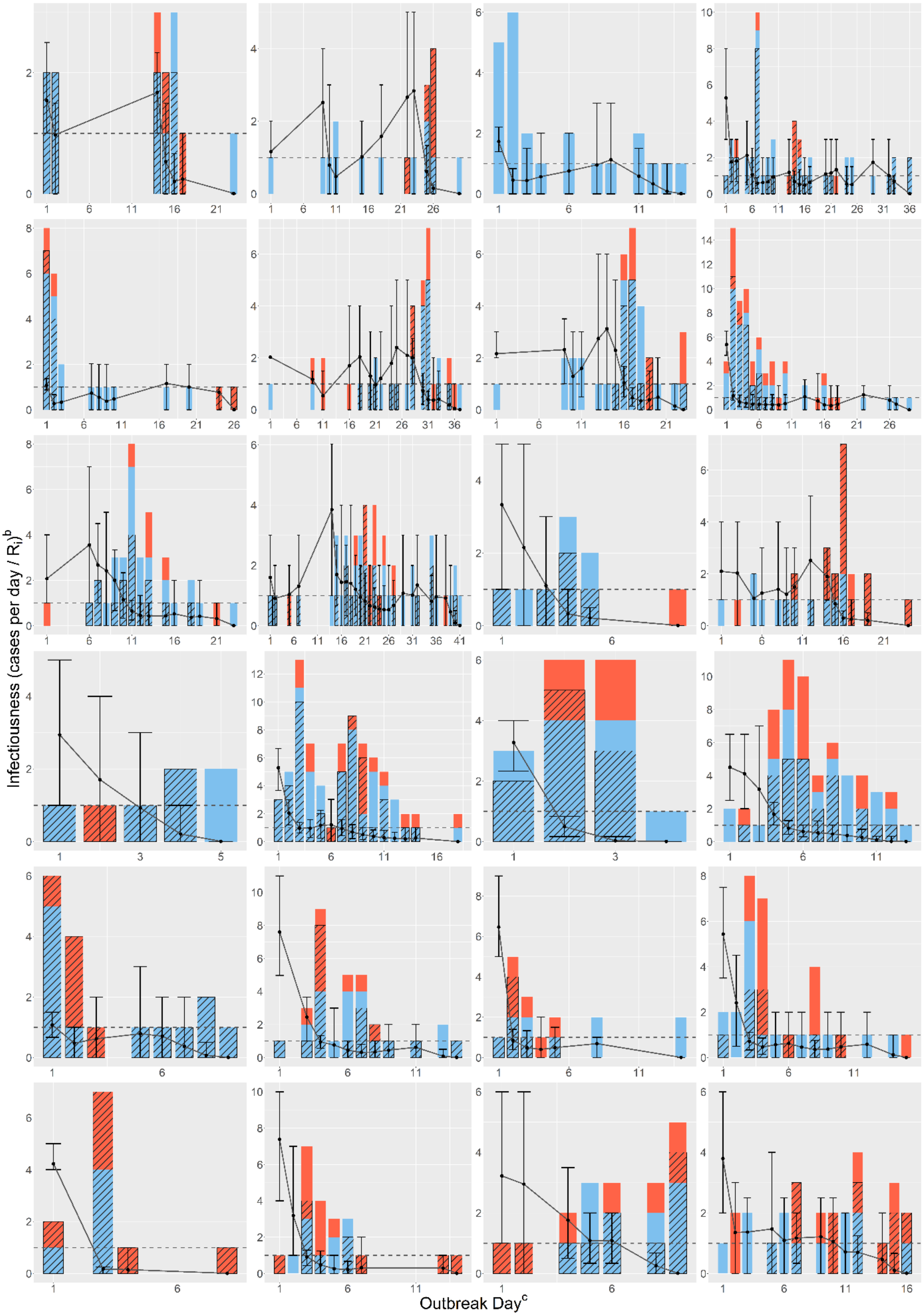

Infectiousness (cases per day / R_i)[b]
Outbreak Day[c]

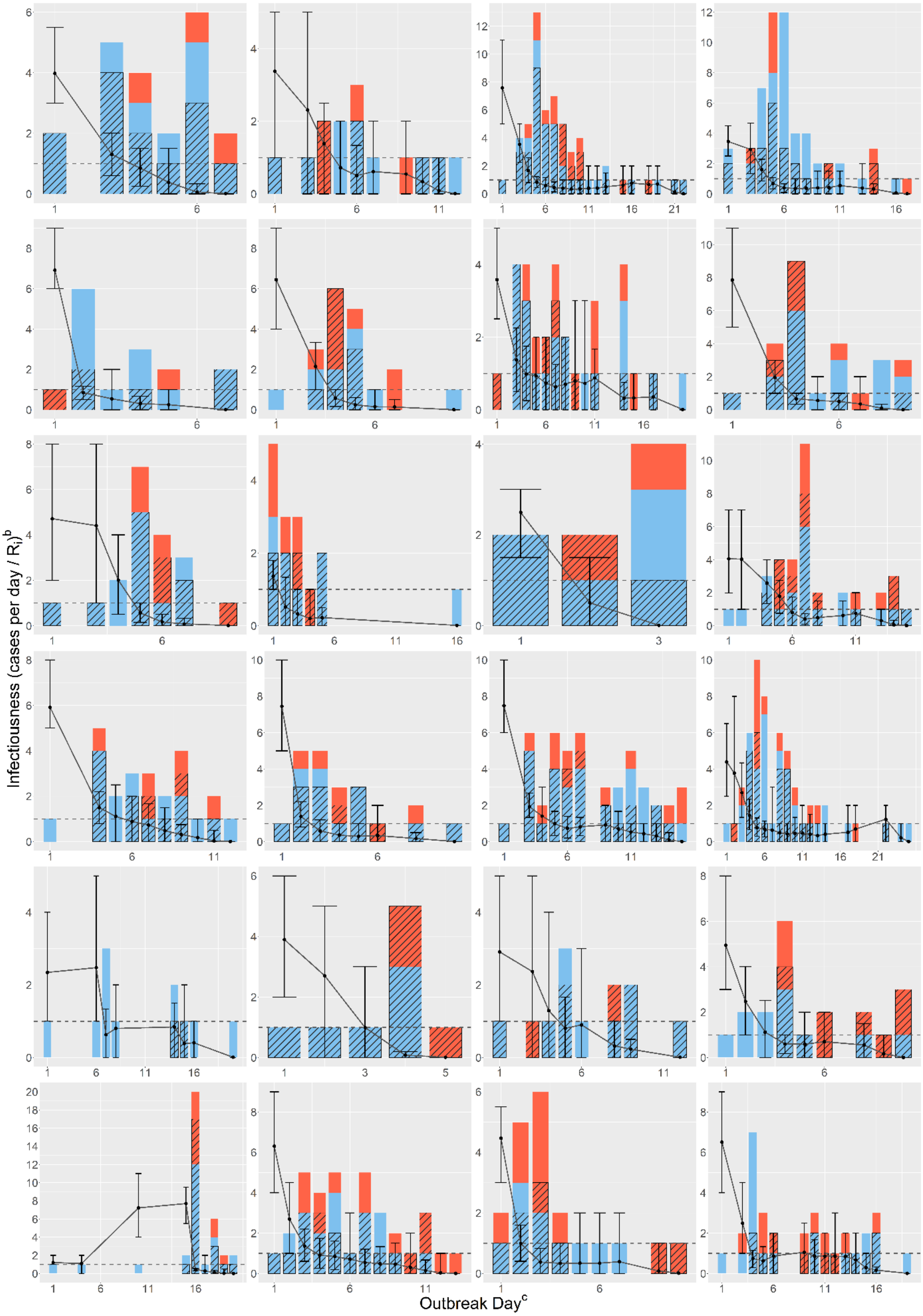

Infectiousness (cases per day / R_i)[b]
Outbreak Day[c]

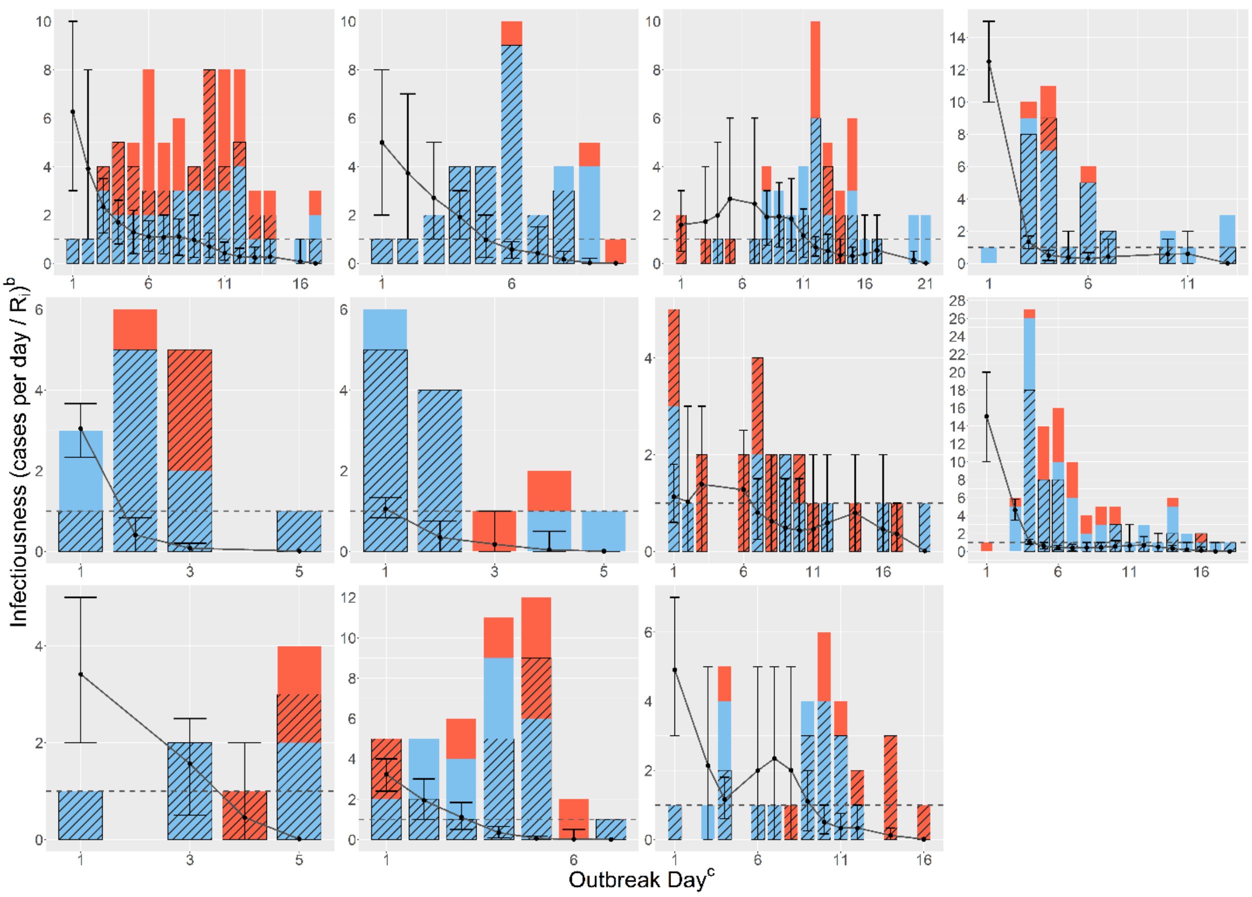

a) In the histogram, blue represents cases who vomited and red represents cases who did not vomit or had missing information on vomiting. Diagonal lines represent resident cases and absence of diagonal lines represent staff cases.

b) Infectiousness describes the number of cases per day (bars) and R_i (point estimates). Dashed horizontal lines signify a R_i of 1, below which an outbreak, on average, cannot be maintained. Note the change in scale for different outbreaks.

c) Outbreak day represents the day into the outbreak, with day 1 corresponding to the first illness onset date.
